# The Association Between Metabolic Status and Risk of Cancer Among Patients With Obesity: Metabolically Healthy Obesity vs. Metabolically Unhealthy Obesity

**DOI:** 10.3389/fnut.2022.783660

**Published:** 2022-02-25

**Authors:** Xiaonan Zheng, Ruilin Peng, Hang Xu, Tianhai Lin, Shi Qiu, Qiang Wei, Lu Yang, Jianzhong Ai

**Affiliations:** ^1^Department of Urology and Institute of Urology of West China Hospital, Sichuan University, Chengdu, China; ^2^Frontiers Science Center for Disease-Related Molecular Network, Institutes for Systems Genetics, West China Hospital, Sichuan University, Chengdu, China; ^3^Department of Radiation Oncology, West China Hospital, Sichuan University, Chengdu, China

**Keywords:** metabolically healthy obesity (MHO), metabolically unhealthy obesity (MUO), risk of cancer, meta-analysis, pan-cancer

## Abstract

**Background:**

Controversial evidence about the association between cancer risk and metabolic status among individuals with obesity has been reported, but pooled data remain absent. This study aims to present pooled data comparing cancer risk between patients with metabolically healthy obesity (MHO) and metabolically unhealthy obesity (MUO).

**Methods:**

The current study systematically searched pieces of literature on January 4, 2021, of prospective cohorts that compare the incidence of cancer between MHO and MUO. The quality of included studies was assessed using Newcastle–Ottawa scale, and publication bias was evaluated using funnel plots.

**Results:**

Eleven high-quality studies were eventually selected. Quantitative analysis indicates that a lower cancer incidence exists for MHO phenotype than that for MUO (odds ratio [OR], 0.71; 95% confidential interval [CI], 0.61–0.84). Consistent outcomes are presented by subgroup analyses, which are grouped by cohort region (western population: [OR, 0.84; 95% CI, 0.75–0.93]; Asian population: [OR, 0.64; 95% CI, 0.54–0.77]); definition of metabolic unhealthiness (≥3 metabolic abnormalities: [OR, 0.62; 95% CI, 0.54–0.71]; ≥1 metabolic abnormality: [OR, 0.76; 95% CI, 0.62–0.94]); and definition of obesity (body mass index (BMI), ≥30 kg/m^2^: [OR, 0.84; 95% CI, 0.73–0.98]; BMI, ≥25 kg/m^2^: [OR, 0.53; 95% CI, 0.52–0.55]).

**Conclusion:**

In conclusion, this study suggests a reduced cancer risk for MHO compared to MUO regardless of population heterogeneity, or the definitions of obesity and metabolic status.

## Highlights

The correlation of metabolic status and cancer risk among individuals with obesity remains controversial. This systematic review and meta-analysis, for the first time, suggests a reduced cancer risk for patients with metabolically healthy obesity compared to those with metabolically unhealthy obesity [OR 0.71, 95% CI 0.61–0.84]. Our findings promote the understanding of the association between metabolic status and cancer risk and also provide further clinical implication of malignancy prevention for individuals with obesity plus metabolic abnormalities.

## Introduction

Obesity, currently prevalent in over 10% of mankind, has tripled since the 1970s and has been a global pandemic for decades' (1, 2). Adequate evidence has reported the increased risk of cardiovascular disease (3), type 2 diabetes (4), cancers (5), and reduced life expectancy (6, 7) for the population with obesity, causing enormous health and socioeconomic burden. Obesity is defined by the World Health Organization as abnormal or excessive fat accumulation. However, observation data have revealed that a proportion of individuals with obesity have less chance of developing metabolic abnormalities and related cardiometabolic diseases (8–11), which implies that the extent of adiposity cannot comprehensively explain the risk of developing obesity-related comorbidities. Therefore, this obesity subgroup is prescribed as metabolically healthy obesity (MHO) (12).

With age- and gender-dependent prevalence of 10–30% (13), MHO is not rare even though the variation of prevalence is high across cohort studies (14, 15), which is mostly caused by the different MHO criteria. Notably, although harmonized criteria have recently been raised (16), no current standard MHO criteria exist. Individuals with obesity are usually referred to as MHO when normal levels of glucose and lipid parameters as well as the absence of hypertension are reported. Otherwise, they are classified as the metabolically unhealthy obesity (MUO) phenotype. For the last two decades, multiple studies have investigated the impact of the metabolic status difference between MHO and MUO on the risk of cardiovascular disease and type 2 diabetes (4, 17).

In recent years, the biological mechanisms underlying obesity, metabolism and tumor have been reported (18–20). Thus, several cohorts have also been conducted to investigate the correlation between metabolic status and cancer risk among individuals with obesity by comparing MHO vs. MUO (21–23). However, controversies of the conclusions from those cohorts still remain, and a lack of collaborative and pooled evidence is noted. Although a previous meta-analysis reported the association between MHO and cancer risk (24), they focus on obesity rather than metabolic status, by comparing MHO with metabolically healthy individuals with normal weight (MHNW). Therefore, this study, for the first time to our knowledge, aims to explore the association between cancer risk and metabolic status among individuals diagnosed with obesity by presenting pooled evidence comparing the cancer incidence between MHO and MUO phenotypes.

## Methods

### Search Strategy

This study followed the Preferred Reporting Items for Systematic Reviews and Meta-Analyses guidelines (25) to systematically search articles that compare the cancer incidence of MHO and MUO in PubMed, Embase, ClinicalTrial.gov, and Cochrane Library Central Register of Controlled Trials database regardless of publication language or date. These terms were used: metabolically healthy obesity, metabolically unhealthy obesity, metabolically healthy obese, MHO, MUO, metabolically obese, metabolically abnormal obesity, metabolically abnormal obese, tumor, cancer, malignancy, and neoplasm.

### Inclusion and Exclusion Criteria

Studies were included if the following inclusion criteria are fulfilled: (1) patients must be divided into different body size-related phenotypes (normal weight or obese), and they were further classified according to their metabolic health status (metabolically healthy or metabolically unhealthy/abnormal); (2) comparative studies of the cancer incidence between MHO and MUO; (3) cohorts focused on malignancies only, excluding benign tumors; and (4) studies providing data that are available for quantitative analysis. The exclusion criteria are as follows: (1) reviews, meta-analysis, case report, or basic science; (2) studies that are MHO-related but without comparison between MHO and MUO were performed; (3) cohorts that do not separate benign and malignant tumors; (4) studies reporting incidence of advanced cancer only rather than any type of cancer; and (5) data not available for quantitative analysis. Two authors have independently selected the articles and resolved the discrepancies through discussion.

### Data Extraction and Quality Assessment

The information including publication year, country, malignant tumor types, cohort size, follow-up duration, cancer incidence rate (per 1,000 person-years), and definition of obesity and metabolic status were collected. The number of events (diagnosis of cancer) and total patients with MHO and MUO phenotypes, respectively, were extracted.

The Newcastle–Ottawa Assessment Scale (NOS) was used to assess the quality of included studies which contains the aspects of selection, comparability, and exposure (26). Studies scored seven or more are ranked as low risk of bias. The funnel plots were used to evaluate publication bias. Publication bias is low when a funnel plot is symmetrical, and the circles representing included studies gathered around the tip of the funnel plot.

### Statistical Analysis

Odds ratio (OR) with 95% confidential interval (CI) was calculated following the number of events of cancer and total patients. A random-effects model was used when the heterogeneity was high. Otherwise, a fixed-effects model was used. Heterogeneity among studies was assessed using *I*^2^ or *Q* tests. An *I*^2^ of >50% or *Q* test reporting *P* < 0.1 indicated that heterogeneity was high. A heterogeneity test was conducted by removing each study in the quantitative analysis to evaluate the possible origins of the heterogeneity. Subgroup analyses were also conducted to investigate the impact of possible confounding (e.g., region and different MHO definitions) by dividing the studies into different subgroups. All the above analyses and plots were conducted using Review Manager (version 5.3).

## Results

### Characteristics of the Included Studies

**Figure 1:**
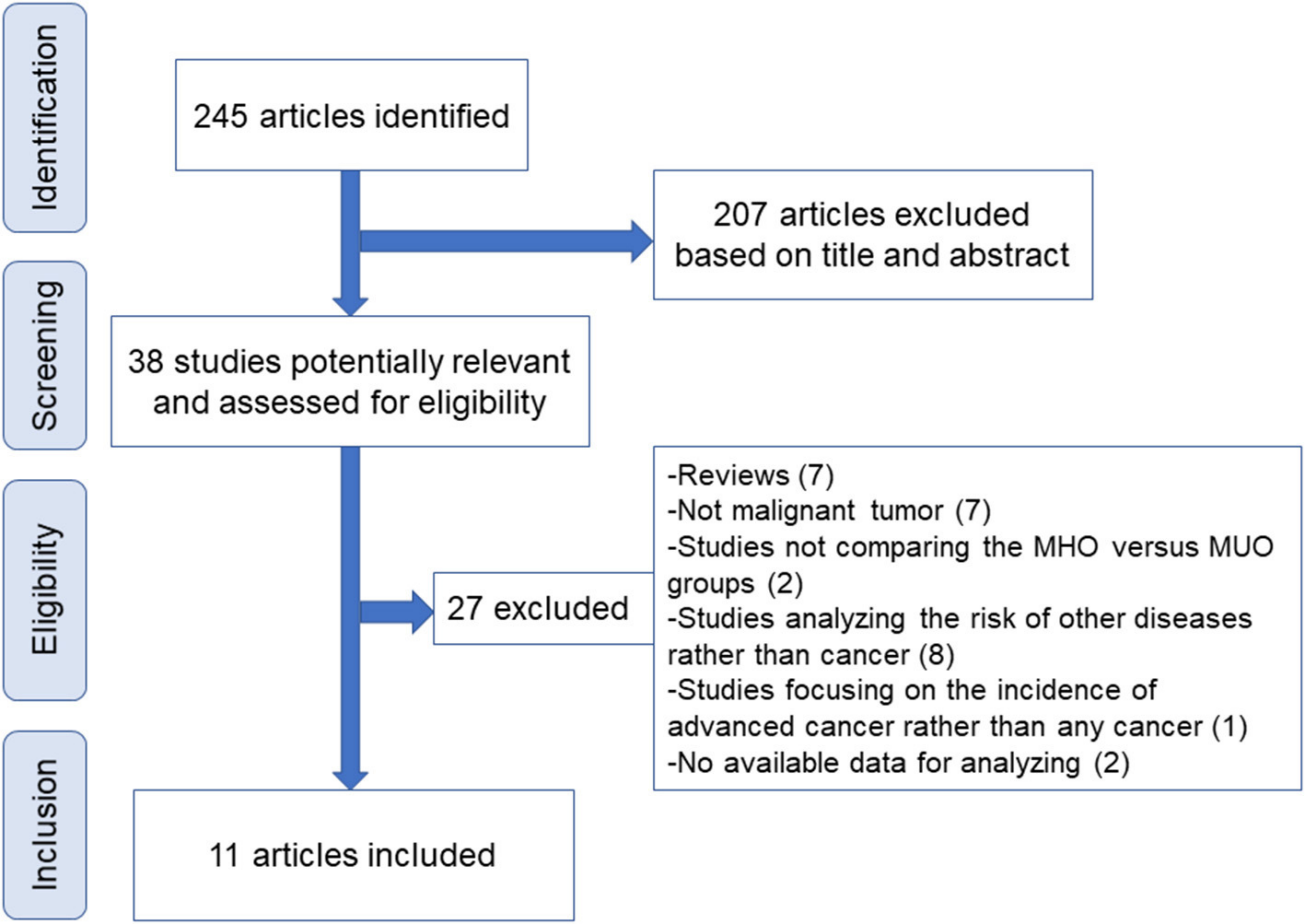
Flowchart of study screening. MHO, metabolically healthy obesity; MUO, metabolically unhealthy obesity.

Figure 1 displays the flowchart of screening eligible studies. Moreover, 245 articles were identified after searching, and 207 were excluded on the basis of titles and abstracts. The other 38 publications were further assessed for eligibility via full-text review, and 11 articles were eventually selected for meta-analysis (21, 22, 27–35). Notably, of the 27 excluded articles, seven studies compared the incidence of colorectal neoplasm (benign tumor included) between MHO and MUO with the overlapped database. One study reported the incidence of pan-cancer using UK Biobank data that do not provide available data for quantitative analysis, whereas another study focused on the incidence of advanced cancer rather than any cancer (Supplementary Table 1).

Table 1 presents the characteristics of the 11 included studies. Five and six studies were conducted in western countries and Asia, respectively. The study by Arnlov reported pan-cancer incidence with a limited number of MHO and MUO patients, and the other 10 studies focused on six types of cancers including breast, colorectal, thyroid, gastric, prostate, and bladder cancers with a maximum of 4,383,392 patients involved. The follow-up duration of those studies generally exceeds 5 years (median) except that the EPIC Study has a median follow-up of 3.7 years. Importantly, the definition of body mass index (BMI) and metabolic status varies among those studies. All of the Asian population-based cohorts consistently define BMI of >25 kg/m^2^ as obesity according to the International Diabetes Federation criteria for the Asian population (1), whereas the western population-based cohorts use BMI of >30 kg/m^2^ to define obesity. However, notably, two western country-based studies by Murphy and Park categorize participants with overweight or obesity as the same group (normal weight vs. overweight and obesity). Metabolic unhealthiness is defined as a diagnosis of three or more metabolic abnormalities regarding triglyceride, high-density lipoprotein cholesterol, fasting glucose, and blood pressure in six studies. However, the other five studies defined metabolic unhealthiness as a diagnosis of one or more metabolic abnormalities. In Supplementary Table 2, the quality of included studies is assessed. All of the studies are ranked as high quality with a NOS score no <7.

**Table 1:** Characteristics of included studies.

**References**	**Region**	**Data source**	**Cancer type**	**Number of patients**		**Follow-up duration**	**Definition of obese**	**Definition of metabolically unhealthy (MU) and metabolically health (MH)**
				**MHO**	**MUO**			
Arnlov et al. (21)	Sweden	Swedish cancer register	Pan-cancer	30	66	Median: 30 years	BMI >30	MU if ≥3 of the following criteria is fulfilled, otherwise MH: • Fasting blood glucose ≥5.6 mmol/l (100 mg/dl) • BP ≥130/85 mmHg or treatment • TG ≥1.7 mmol/l (150 mg/dl) • High density lipoprotein cholesterol <1.04 mmol/l (40 mg/dl) • BMI ≥29.4 kg/m^2^
Murphy et al. (22)	Europe	EPIC study	Colorectal cancer	214	737	Median: 3.7 years	BMI ≥25 (overweight, obese)	C-peptide concentration tertile cut-points: 2.96 ng/ml and 4.74 ng/ml, MHO if below the first tertile of C-peptide and MUO if above the first tertile
Kabat et al. (28)	USA	Women's health initiative memory study	Breast cancer	3,347	4,902	15 years (Overall)	BMI ≥30	MU if ≥3 of the following criteria is fulfilled, otherwise MH: WC ≥88 cm, TG ≥150 mg/dL, HDL-C <50 mg/dL, glucose ≥100 mg/dL, and systolic/diastolic BP ≥130/85 mmHg or treatment for hypertension
Park et al. (29)	USA	Sister study	Breast cancer	6,014	20,966	Mean: 6.4 years	BMI ≥25 (overweight, obese)	MU if ≥1 of the following criteria is fulfilled, otherwise MH: WC ≥88 cm, TG ≥150 mg/dL, HDL-C <50 mg/dL, glucose ≥100 mg/dL, and systolic/diastolic BP ≥130/85 mmHg or treatment for hypertension
Kabat et al. (30)	USA	Women's health initiative memory study	Colorectal cancer	4,038	4,931	15 years (Overall)	BMI ≥30	MU if ≥3 of the following criteria is fulfilled, otherwise MH: WC ≥88 cm, TG ≥150 mg/dL, HDL-C <50 mg/dL, glucose ≥100 mg/dL, and systolic/diastolic BP ≥130/85 mmHg or treatment for hypertension
Kwon et al. (34)	Korea	Kangbuk samsung health study	Thyroid cancer	15,402	58,884	Median: 5.3 years	BMI ≥25	MU if ≥1 of the following criteria is fulfilled, otherwise MH: Fasting glucose level ≥ 100 mg/dL or current use of glucose-lowering agents, BP ≥ 130/85 mmHg or current use of BP-lowering agents, elevated TG level (≥ 150 mg/dL) or current use of lipid-lowering agents, low HDL-C (< 40 mg/dl in men or < 50 mg/dl in women), or insulin resistance, defined as an HOMA-IR score ≥ 2.5
Hashimoto and Hamaguchi (35)	Japan	NAGALA study	Gastric cancer	653	3,425	Median: 5.5 years	BMI ≥25	MU if ≥1 of the following criteria is fulfilled, otherwise MH: Impaired fasting plasma glucose and/or diabetes was defined as fasting plasma glucose > 5.6 mmol/L and/or current medical treatment. Hypertension was defined as systolic BP > 130 mmHg and/or diastolic BP > 85 mmHg or current medical treatment. Elevated TG were defined as TG > 1.7 mmol/L or treatment for hyperlipidemia. Low HDL-cholesterol was defined as <1.0 mmol/L in men and < 1.3 mmol/L in women.
Cho et al. (31)	Korea	NHIS-HEALS	Colorectal cancer	28,557	86,238	2009–2015	BMI ≥25	MU if ≥1 of the following criteria is fulfilled, otherwise MH: (1) systolic BP ≥130 mmHg and/or diastolic BP ≥85 mmHg and/or taking antihypertensive medications; (2) TG level ≥150 mg/dl and/or taking lipid-lowering medications; (3) FPG level ≥100 mg/dl and/or taking antidiabetic medications; and (4) HDL-C levels <40 mg/dl in men and <50 mg/dl in women
Chung et al. (32)	Korea	NHIS-HEALS	Pancreatic cancer	65,983	54,349	median: 6.1 years	BMI ≥25	MU if ≥3 of the following criteria is fulfilled, otherwise MH: Fasting glucose levels ≥5.6 mmol/L (100 mg/dL) or the current use of glucose-lowering agents under the ICD-10 codes E10–E14; BP ≥130/85 mmHg or the use of antihypertensive agents under the ICD-10 codes I10–15; serum TG levels ≥1.7 mmol/L (≥150 mg/dL) or the current use of lipid-lowering agents under the ICD-10 code E78; HDL-C levels <1.0 mmol/L (40 mg/dL) in men or <1.3 mmol/L (50 mg/dL) in women or the current use of lipid-lowering agents under the ICD-10 code E78; and (WC) WC >90 cm for men or ≥85 cm for women, based on the International Diabetes Federation criteria for the Asian population.
Kim (27)	Korea	NHC databases	Bladder cancer	2,313,991	2,069,401	Median: 5.4 years	BMI ≥25	MU if ≥3 of the following criteria is fulfilled, otherwise MH: TG level ≥150 mg/dL, HDL-C level <40 mg/dL, fasting glucose level ≥100 mg/dL (or taking anti-diabetic medications), BP ≥ 130/85 mmHg (or taking antihypertensive drugs), or WC ≥ 90 cm, according to the Asian-specific waist circumference cut-off
Kim (33)	Korea	NHC database	Prostate cancer	2,312,838	2,067,004	Median: 5.4 years	BMI ≥25	MU if ≥3 of the following criteria is met, otherwise MH: TG ≥ 150 mg/dL, HDL-C < 40 mg/dL, fasting glucose ≥ 100 mg/dL, BP ≥ 130/85 mmHg (or taking antihypertensive drug treatment), or WC > 90 cm, according to the International Diabetes Federation criteria for Asian countries.

*MHO, metabolically healthy obesity; MUO, metabolically unhealthy obesity; BMI, body mass index (kg/m^2^); BP, blood pressure; HDL-C, high-density lipoprotein cholesterol; TG, triglycerides; WC, waist circumference*.

### Comparison of Cancer Incidence Between MHO and MUO Phenotypes

**Figure 2:**
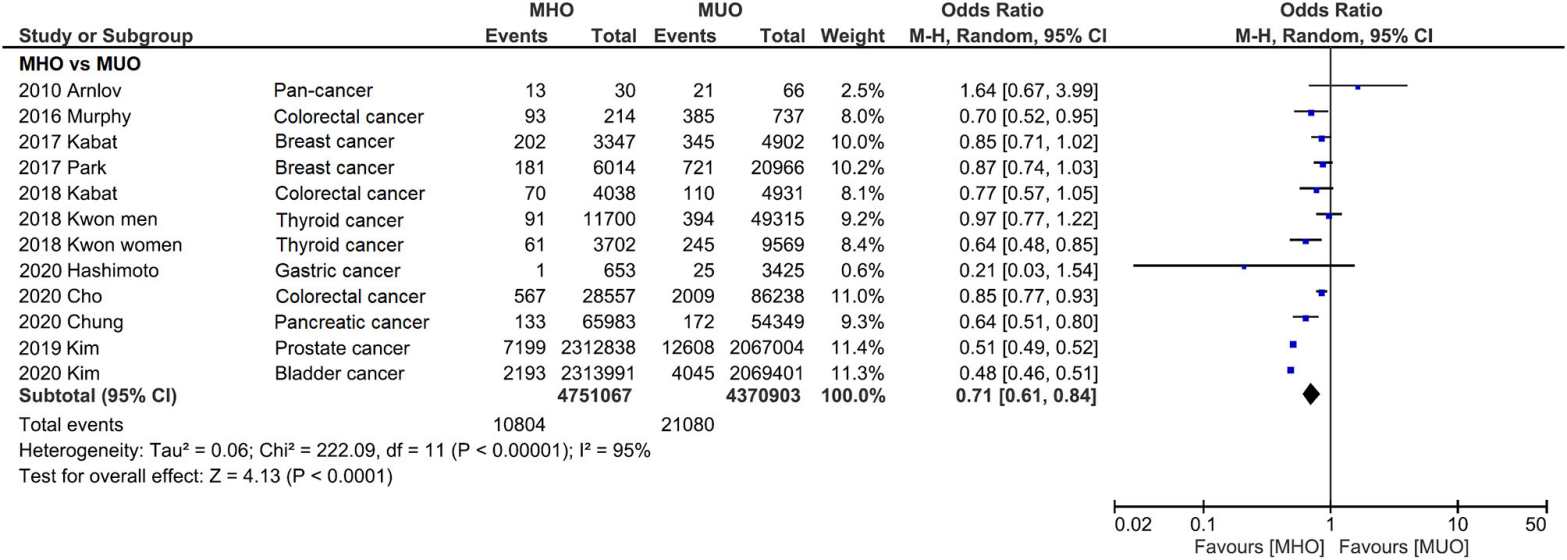
Forest plot that compares cancer incidence between metabolically healthy obesity and metabolically unhealthy obesity. Cancer incidence is significantly lower for metabolically healthy obesity.

In Figure 2, the incidence of cancer is compared between MHO and MUO phenotypes. Except for Arnlov's study, all the other studies show favorable MHO outcomes, although statistical significance is not reached in five studies. Notably, because Kown reported the data of men and women (34), those data were also separately presented in the present study.

**Figure 3:**
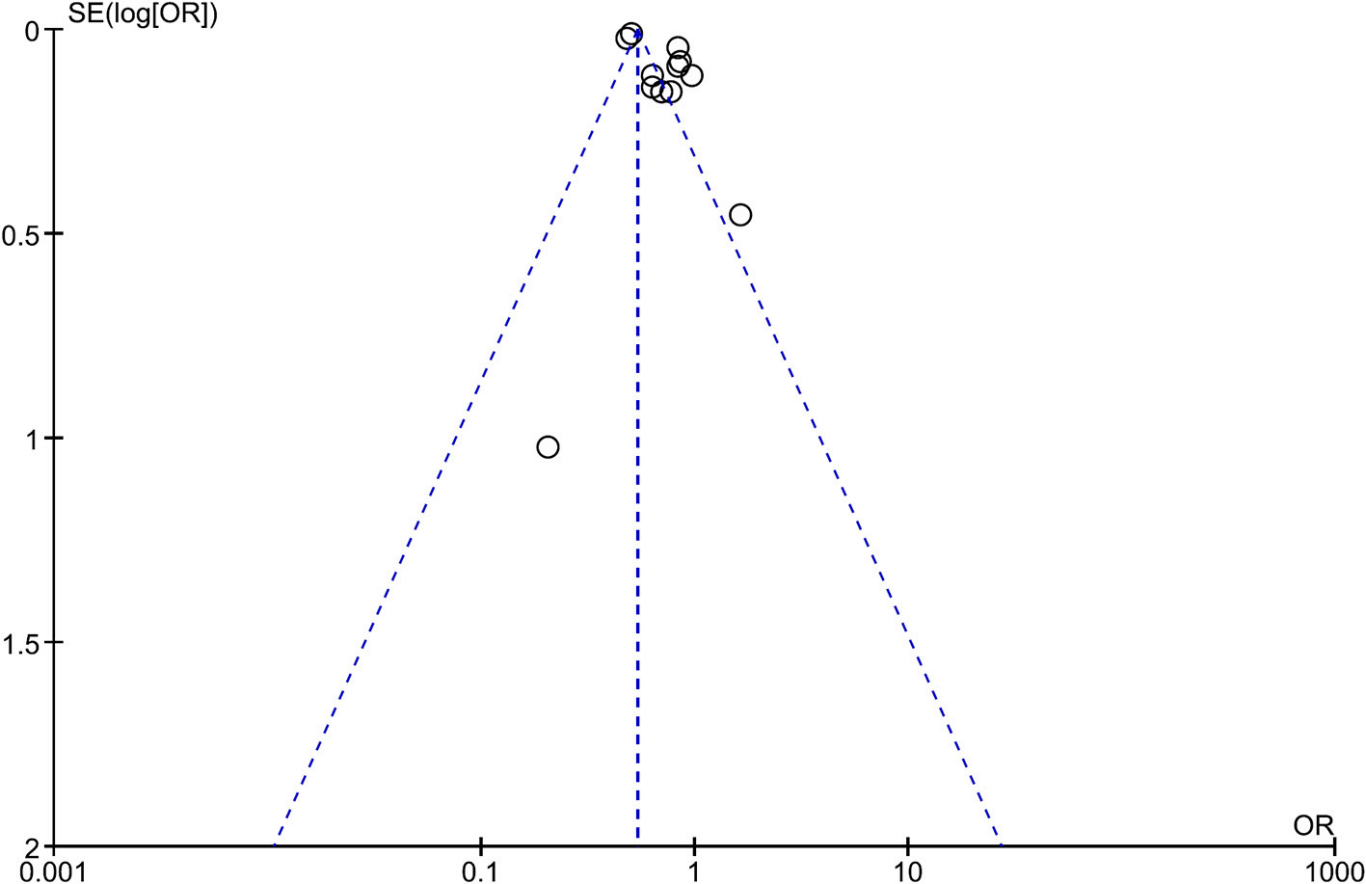
Funnel plot of analysis comparing cancer incidence between metabolically healthy obesity and metabolically unhealthy obesity.

Consistently, pooled outcome indicates that cancer incidence is 29% lower in MHO than that in MUO (OR, 0.71; 95% CI, 0.61–0.84), despite the interstudy heterogeneity remaining high (*I*^2^ = 95%). The funnel plot indicates that the publication bias of this quantitative analysis is insignificant with a symmetrical funnel plot, and *P* value of 0.671 and 0.115 for Egger's test and Begg's test, respectively (Figure 3 and Supplementary Figure 1). Further analyses excluded different studies in quantitative analysis to rule out the reasons for heterogeneity. Supplementary Figure 2 shows that the heterogeneity drops to *I*^2^ = 45% and *I*^2^ = 28% after removing two (27, 33) and three (27, 32, 33) studies.

**Figure 4:**
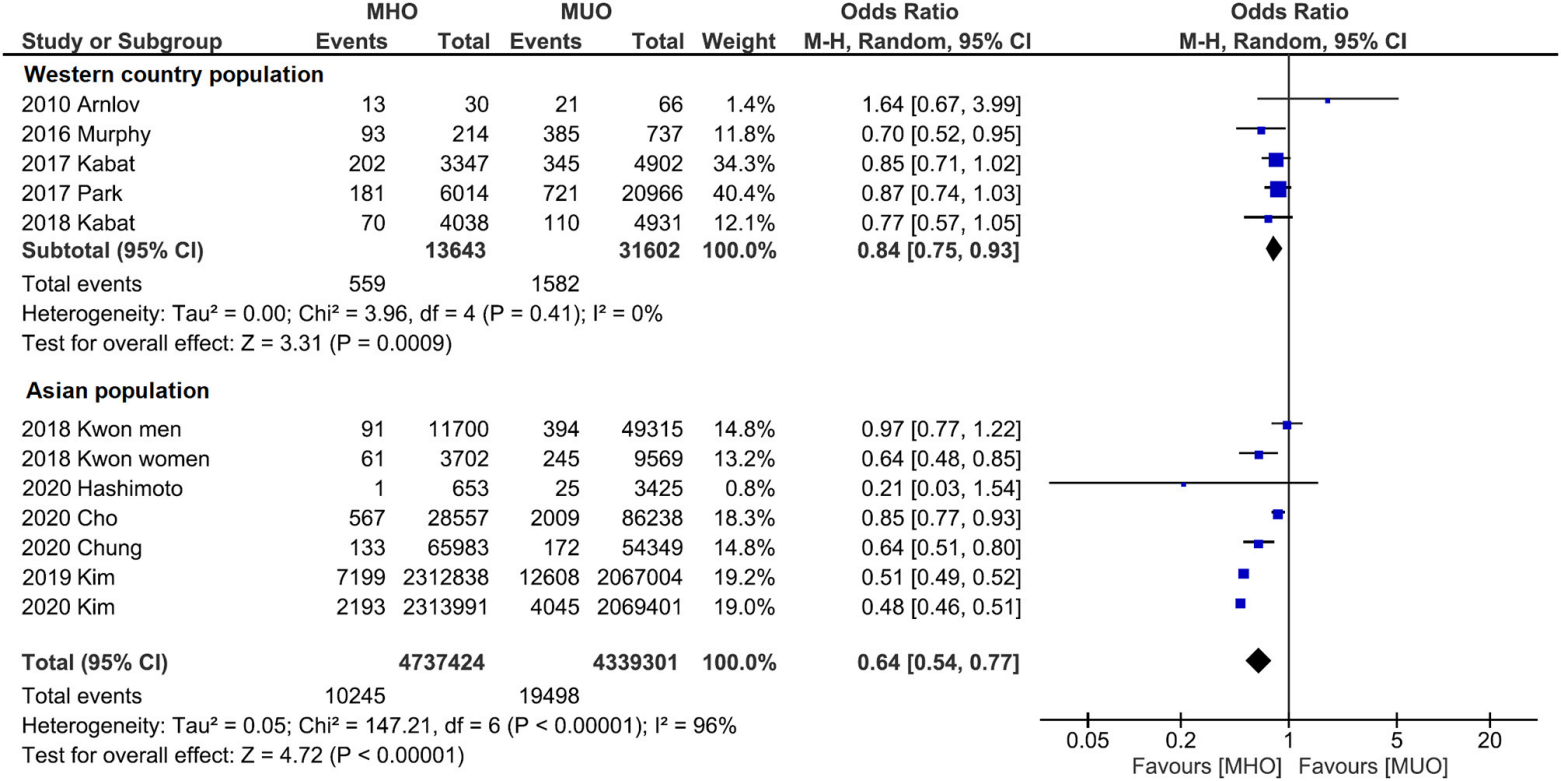
Subgroup analysis grouped by the region of cohorts. Lower cancer incidence for metabolically healthy obesity phenotype is found in both western population and Asian population.

**Figure 5:**
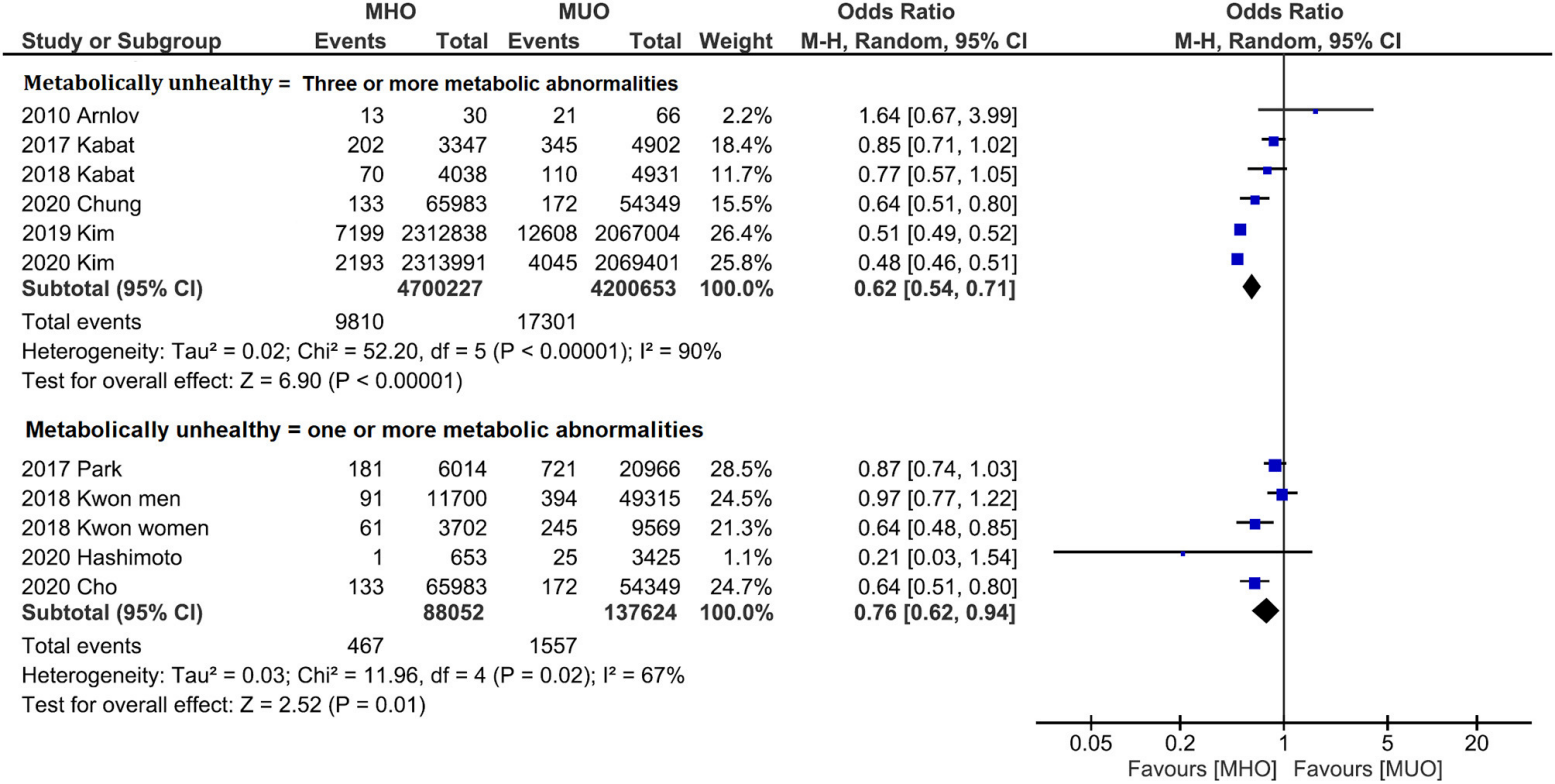
Subgroup analysis grouped by the definition of metabolically status. Lower cancer incidence for metabolically healthy obesity phenotype is found in both studies defining three or more metabolic abnormalities as metabolically unhealthy and studies defining one or more metabolic abnormalities as metabolically unhealthy.

Subgroup analyses were also conducted to reveal the potential confounding effects of the present study findings. Figure 4 displays the outcome categorized by population. Quantitative analyses of both western (OR, 0.84; 95% CI, 0.75–0.93; *I*^2^ = 0%) and Asian (OR, 0.64; 95% CI, 0.54–0.77; *I*^2^ = 96%) populations indicate that MHO phenotype has lower cancer incidence. Likewise, subgroup analysis by the definition of metabolic unhealthiness shows favorable MHO evidence (Figure 5). Pooled OR (MHO versus MUO) is 0.62 (95% CI, 0.54–0.71; *I*^2^ = 90%) and 0.76 (95% CI, 0.62–0.94; *I*^2^ = 67%) for studies defining metabolic unhealthiness as three or more and one or more metabolic abnormalities, respectively. Moreover, the present study further conducted subgroup analysis according to the definition of obesity (Supplementary Figure 3) and MHO phenotype has a lower incidence of cancer in two subgroups either defining BMI of ≥30 kg/m^2^ as obesity (OR, 0.84; 95% CI, 0.73–0.98; *I*^2^ = 19%) or BMI of ≥25 kg/m^2^ as obesity (OR, 0.53; 95% CI, 0.52–0.55; *I*^2^ = 96%).

The present study also compared MHNW with MHO, MUO, and metabolically unhealthy normal weight (MUNW) to more comprehensively investigate the impact of different phenotypes on cancer incidence. Moreover, MHNW phenotype has consistently lower cancer incidence compared with MUNW (OR, 1.19; 95% CI, 1.13–1.25; *I*^2^ = 55%), MHO (OR, 1.29; 95% CI, 1.23–1.35; *I*^2^ = 56%), and MUO (OR, 1.09; 95% CI, 1.07–1.11; *I*^2^ = 26%; Supplementary Figure 4).

## Discussion

The investigation of the impact of MHO on cardiovascular diseases and type 2 diabetes has been an ongoing effort ever since the MHO concept was raised in the 1950's by Dr. Jean Vague' (12). In recent years, researchers have started to focus on the correlation between MHO and the risk of cancer. Although a previous meta-analysis compares the risk of cancer between MHO and MHNW (24), their study is mostly about the impact of obesity on the risk of cancer among patients without metabolic abnormalities. Compared to metabolically healthy population with normal weight, they claimed, MHO had a significantly increased chance of developing cancer (OR 1.14, 95% CI 1.05–1.23), which was independent from the modification by age, sex, ethnicity, smoking, sample size or length of follow-up. Hence, their study found an increased risk of cancer related to obesity itself. Nevertheless, evidence that presents the correlation between metabolic status and cancer risk among patients with obesity is still lacking. The present study, therefore, investigated cancer incidence between MHO and its comparative phenotype—MUO. Our meta-analysis indicates that there is a reduced risk of cancer for MHO phenotype compared with MUO phenotype. Subgroup analyses show consistent outcomes after cohorts from different regions or using different definitions of obesity and metabolic status are distinguished.

The impact of MHO on diseases (e.g., cardiovascular diseases and cancer) has remained debated. The disagreement may be originated from multiple confounding. Ununified MHO definition is believed to be an important confounding factor that limits the interpretation of relevant studies. Although the MHO concept was raised decades ago, more than 30 different definitions of metabolic health have been used (36). Moreover, the heterogeneity among those definitions may lead to a significantly different MHO prevalence. For instance, Bluher reviewed the MHO prevalence in the National Health and Nutrition Examination Survey III program (13) and found that 40% of the participants are classified as MHO using the National Cholesterol Education Program Adult Treatment Panel III criteria (37). However, the MHO proportion drops to 20% when more strict insulin sensitivity parameter cutoffs are used (38). Similarly, a Chinese cohort reported that the MHO prevalence varies between 4.2 and 13.6% when different definitions are used (14). Thus, the present study distinguished the definition of metabolic health and obesity to perform subgroup analyses. Notably, although consistent outcomes between subgroups were found in the present analyses, the need for standardized MHO criteria should still be addressed. Additionally, subgroup analysis was also conducted based on the regions of the included cohorts, given that the significant regional difference of MHO prevalence that was previously reported (39, 40). MHO was also found to have a lower risk of cancer compared with MUO in either western or Asian countries, which necessitates multiregional studies in the future to compare the risk of cancer between MHO and MUO.

The limitation of this study is that the analysis of the impact of demographic characteristics (e.g., age and gender) on the association between MHO and cancer incidence is absent because of the lack of available data from the included studies. Previous evidence shows that MHO persistence is correlated with younger age and consistently decreases with increasing age (40). Moreover, the general variation of MHO prevalence between males and females is also indicated across a collaborative study of 10 European cohorts (15). Nevertheless, Lin et al. conducted a metaregression and revealed that age and gender (also ethnicity and smoking status) do not significantly affect the cancer risk among individuals with MHO (24), although the study by Kwon indicates an incidence that is twice higher in rate (per 1,000 person-years) of thyroid cancer in females than in males with MHO (34). However, the incidence rate of cancer between females and males should be appropriately interpreted. Most of the cohorts included in the present study emphasized the incidence of a single cancer type for patients with MHO. However, the variation of cancer incidence could be substantial among different cancer types. For instance, some types of cancer can be hormone-related (e.g., breast and thyroid cancers), and the hormone level between genders is distinct. Some cancer types have even been well-recognized to be more prevalent between gender [e.g., bladder cancer, whose incidence of men to women is roughly 4:1 (41)]. This potential bias, therefore, warrants future studies to investigate the association more comprehensively between cancer risk and MHO individuals. Analyzing pan-cancer risk using a similar cohort with adequate follow-up duration would be a feasible strategy. Additionally, the pan-cancer analysis within a single cohort may also more objectively and accurately present the true cancer incidence rate of individuals with MHO. Previously, although Arnlov and Cao conducted pan-cancer analysis among MHO individuals, their studies either have a small sample size (<100 participants with MHO and MUO individuals combined) (21) or do not provide cancer incidence rate of MHO individuals (23).

In terms of the biological differences, MHO is believed to have greater insulin sensitivity, better insulin secretion, normal inflammatory markers and normal adipose tissue function, while MUO is more likely to show insulin resistance, higher markers of inflammation and adipose tissue dysfunction (13). Insulin sensitive MHO is associated with less immune cell infiltration into visceral fat depots, lower mean adipocyte size and a favorable adipokine secretion pattern, while a pro-inflammatory, diabetogenic and atherogenic secretion pattern may contribute to the development of MUO (42). Another critical debate over MHO is whether it represents a *stable condition*, which may also influence the interpretation of the present findings. Bluher's review proposed that individuals in long-term obesity treatment programs may undergo cycles of weight loss and regain accompanied by changing their phenotype from MUO to MHO and back to MUO (13). Multiple longitude studies have also demonstrated that an proportion of MHO defined at the baseline will undergo the transition to MUO when the follow-up is long enough, although this transition is not necessarily a one-way road (39, 43–45). The gender difference behind MHO-MUO transition still maintains controversial (40, 46), and the lower MHO prevalence in postmenopausal than in premenopausal women suggests that changes in sex hormones may promote this transition (47).

This study is believed to be the first systematic review and meta-analysis combining the evidence comparing the risk of cancer between MHO and MUO. Another strength of the present study is the subgroup analyses investigating the influence of potential confounding on the outcomes. Nevertheless, limitations need to be indicated. Besides the aforementioned potential bias caused by ununified MHO definitions, demographic characteristics (e.g., age, gender, and region), study design flaw, and transition between MHO and MUO, the definition of obesity (waist circumstance or BMI) is not discussed in the present study. Moreover, the heterogeneity is substantial in the present analyses, and some of the included studies are using different big registry cohorts from the same country, which may also produce overlapping data. Last but not the least, although all of the included studies are prospective and of high-quality, randomized-controlled trials on this topic are not feasible (24) and limit the causality investigation.

In conclusion, this study suggests a reduced cancer risk for MHO compared to MUO regardless of population heterogeneity, or the definitions of obesity and metabolic status. In the future, several key factors in designing the studies should be paid attention to. First, a standardized concept of MHO should be employed across the studies to avoid unnecessary bias. Second, the multicenter prospective observational cohorts should be conducted across different regions to reduce the heterogeneity of cancer risk among different races. Moreover, the future study should avoid reporting the incidence of a single type of cancer but reporting all the types of cancer that are observed. Importantly, the proportion of female and male participants should be balanced so that the bias caused by gender and hormone levels could be much avoided. Lastly, experimental assays are required to further explore the underlying mechanism between metabolic status and cancer risk among patients with obesity.

## Data Availability Statement

The raw data supporting the conclusions of this article will be made available by the authors, without undue reservation.

## Author Contributions

XZ, JA, and LY: conceptualization. XZ, HX, RP, TL, and SQ: search and screening. XZ, HX, RP, and TL: data extraction, data validation, and quality assessment. XZ, HX, and RP: statistical analysis, interpreted the results, and writing (original draft preparation). XZ, JA, LY, and QW: writing (review and editing). All authors have approved the final version and submission.

## Funding

This work was supported by the National Natural Science Foundation of China (82070784 and 81702536 to JA; 82170785 and 81974099 to LY). China Postdoctoral Science Foundation (No. 2021M692306 to XZ) and Post-Doctor Research Project, West China Hospital, Sichuan University (No. 2021HXBH025 to XZ).

## Conflict of Interest

The authors declare that the research was conducted in the absence of any commercial or financial relationships that could be construed as a potential conflict of interest.

## Publisher's Note

All claims expressed in this article are solely those of the authors and do not necessarily represent those of their affiliated organizations, or those of the publisher, the editors and the reviewers. Any product that may be evaluated in this article, or claim that may be made by its manufacturer, is not guaranteed or endorsed by the publisher.

## Abbreviations

MHO: metabolically healthy obesity
MUO: metabolically unhealthy obesity
MHNW: metabolically healthy normal weight
OR: odds ratio
CI: confidential interval
BMI: body mass index.
