# Supplementary material for: The Association Between Metabolic Status and Risk of Cancer Among Patients With Obesity: Metabolically Healthy Obesity vs. Metabolically Unhealthy Obesity
Source: Front Nutr. 2022 Feb 25;9:783660. doi: 10.3389/fnut.2022.783660 (PMC8914254; doi:10.3389/fnut.2022.783660)

Table of Contents

[Table s1. Characteristics of nine excluded studies comparing the incidence of cancer between MHO and MUO. 2](#_Toc93665416)

[Table s2. Newcastle-Ottawa Scale assessing the quality of included studies. 6](#_Toc93665417)

[Table s3. Incidence rate of cancer in selected studies. 7](#_Toc93665418)

[Figure s1. Publication bias assessment by Egger’s test and Begg’s test. 8](#_Toc93665419)

[Figure s2. Heterogeneity test to reveal the cause of heterogeneity by excluding certain studies 8](#_Toc93665420)

[Figure s4. Forest plots comparing the incidence of cancer between different groups (MHO, MUO and MUNW) versus MHNW using the reported odds ratios 10](#_Toc93665421)

[Figure s5. Funnel plot for studies comparing the incidence of cancer between MHO and MHNW groups. 11](#_Toc93665422)

Figure s6. Funnel plot for studies comparing the incidence of cancer between MUO and MHNW groups……………………………………………………………………………………………...11

Figure s7. Funnel plot for studies comparing the incidence of cancer between MUNW and MHNW groups..…………………………………………………………………………………...11

# ****Table s1. Characteristics of nine excluded studies comparing the incidence of cancer between MHO and MUO.****

| Study | Region | Data source | Cancer type | Study size | | Follow-up duration | Definition of obese | Definition of metabolically unhealthy (MU) and metabolically health (MH) |
| --- | --- | --- | --- | --- | --- | --- | --- | --- |
|  |  |  |  | MHO | MUO |  |  |  |
| 2020 Cao | British | UK Biobank | Pan-Cancer | 26094 | 70079 | median: 7.8 years | BMI >30 | MU if ≥2 of the following criteria is fulfilled, otherwise MH: (1) elevated BP, defined as a systolic BP ≥ 130 and/or a diastolic BP ≥ 85 mmHg and/or the use of antihypertensive medication at baseline,  (2) hypertriglyceridemia, defined as TG ≥1.7 mmol/L (150 mg/dL) or current use of lipid-lowering medication at baseline,  (3) low HDL-C, defined as <1.0 mmol/L (40 mg/dL) for men and <1.3 mmol/L (50 mg/dL) for women,  (4) hyperglycaemia, defined as FBG ≥5.6 mmol/L or use of medications for diabetes at baseline (e.g., insulin or oral antidiabetic medications) |
| 2019 Kim | Korea | The Kangbuk Samsung Health Study | Metachronous Colorectal Neoplasia | 351 | 3333 | median: 3.5 years | BMI >25 | MU if ≥1 of the following criteria is fulfilled, otherwise MH:  (1) abdominal obesity (WC ≥ 90 cm in men and ≥ 85 cm in women, the proposed cutoff values for Asians)28,  (2) elevated FBG levels ≥ 100 mg/dL or use of glucose-lowering medications,  (3) elevated BP (≥ 130-mmHg systolic and ≥ 85-mmHg diastolic) or use of antihypertensive drugs,  (4) elevated TG levels (≥ 150 mg/dL or specific treatment for this lipid abnormality),  (5) and reduced HDL-C levels (< 40 mg/dL in men and < 50 mg/dL in women). |
| 2018 Kim | Korea | The Kangbuk Samsung Health Study | Colorectal Neoplasia | 6789 | 39477 | 11 years  (overall) | BMI >25 | MU if ≥1 of the following criteria is fulfilled, otherwise MH:  Abdominal obesity (WC ≥90 cm in men and ≥85 cm in women, the proposed cutoff values for Asians), elevated FBG levels ≥100 mg/dL or hemoglobin A_1c_≥6.5, or use of glucose-lowering medications, elevated BG (≥130 mm Hg systolic, ≥85 mm Hg diastolic) or use of antihypertensive drugs, elevated TG levels (≥150 mg/dL or specific treatment for this lipid abnormality), and reduced HDL-C levels (<40 mg/dL in men and <50 mg/dL in women). |
| 2017 Kim | Korea | the Center for Health Promotion of Samsung Medical Center | Colorectal Neoplasia | 707 | 2612 | 7 years  (overall) | BMI ≥ 25 | MU if ≥1 of the following criteria is fulfilled, otherwise MH:  (1) high serum TG, defined as ≥ 150 mg/dL (1.7mmol/L) or drug treatment for this lipid abnormality,  (2) low HDL-C, defined as≤40 mg/dL (1.0 mmol/L) for men and≤50 mg/dL (1.3 mmol/L) for women or drug treatment for this lipid abnormality,  (3) high BP, defined as BP≥130/85mmHg or drug treatment for previously diagnosed hypertension,  (4) high FBG, defined as>100 mg/dL (5.6 mmol/L) or drug treatment for previously diagnosed diabetes. |
| 2017 Shin | Korea | the National Health Insurance System (NHIS) | Colorectal Neoplasia | 450714 | 554997 | 5 years  (overall) | BMI >25 | The presence of diabetes mellitus was defined as ≥1 claim per year for the prescription of oral hyperglycemics or insulin medication based on the International Classification of Disease, 10^th^ Revision (ICD–10), codes E10 to E14, or a FBG ≥7 mmol/L (obtained from the health examination database). The presence of hypertension was defined as the presence of ≥1 claim per year for the prescription of an antihypertensive agent according to the ICD-10 codes I10 to I15 or systolic/diastolic BP ≥140/90 mmHg. The presence of dyslipidemia was defined as the presence of ≥1 claim per year for the prescription of an antihyperlipidemic agent according to the ICD-10 code E78 or total cholesterol ≥6.21 mmol/L (obtained from the health examination database). Among the subjects with a BMI >25 kg/m^2^, those who developed >1 of the metabolic diseases above in the index year were considered MUO individuals, whereas those without the 3 metabolic diseases were considered MHO individuals. |
| 2017 Ko | Korea | Health Promotion Center of Seoul St. Mary’s Hospital | Colorectal Neoplasms | 1538 | 1855 | 5 years  (overall) | BMI ≥25 | MU if ≥2 of the following criteria is fulfilled, otherwise MH:  (1) systolic BP ≥130 mmHg or diastolic blood pressure ≥85 mmHg or use of antihypertensive medication,  (2) TG levels ≥1.7 mmol/L or use of lipid-lowering drugs,  (3) FBG ≥100 mg/dL or use of anti-diabetes medication,  (4) HDL-C levels <1.03 mmol/L in men and <1.29 mmol/L in women,  (5) HOMA-IR greater than the 90th percentile in our population (≥3.06). |
| 2016 Kim | Korea | Kangbuk Samsung Hospital | Colorectal Neoplasia | 2626 | 21030 | 10 years  (overall) | BMI ≥25 | Subjects with no MetA and who were only obese were categorized as MHO, and subjects with 1 or more MetA and who were obese were categorized as metabolically abnormal obese. MetA was defined as follows:  (1) hypertriglycerides (TG ≥150 mg/dL or specific treatment for this lipid abnormality),  (2) HDL-C abnormality (high-density lipoprotein-cholesterol <40 mg/dL for men and<50 mg/dL for women),  (3) hypertension-related factor (elevated BP ≥130/85 mm Hg or use of antihypertensive medications),  (4) diabetes mellitus–related factor (FBG ≥100 mg/dL or hemoglobin A_1c_ ≥6.5%, or use of diabetes medications). |
| 2020 Cho | Korea | Korean National Health Insurance Service-National Health Screening Cohort | colorectal Neoplasia | 28557 | 86238 | 6 years  (overall) | BMI >25 | Metabolic health was defined as having none or one of the following risk factors:  (1) systolic BP ≥130 mmHg and/or diastolic BP ≥85 mmHg and/or taking antihypertensive medications,  (2) TG level ≥150 mg/dl and/or taking lipid-lowering medications,  (3) FPG level ≥100 mg/dl and/or taking antidiabetic medications,  (4) HDL-C levels <40 mg/dl in men and <50 mg/dl in women. |

MHO = Metabolically Healthy Obesity; MUO = Metabolically Unhealthy Obesity; BMI = Body Mass Index (kg/m^2^); BP = Blood Pressure; HDL-C =High-Density Lipoprotein cholesterol; TG = Triglycerides; WC = Waist Circumference; TBG = Fasting Blood Glucose

# ****Table s2. Newcastle-Ottawa Scale assessing the quality of included studies.****

| Study | Selection | | | | comparability | | Exposure | | | Score |
| --- | --- | --- | --- | --- | --- | --- | --- | --- | --- | --- |
|  | REC | SNEC | AE | DO | SC | AF | AO | FU | AFU |  |
| 2010 Arnlov | * | * | * | * | * | * | * | * | * | 9 |
| 2016 Murphy | * | * | * | * | * | * | * | / | * | 8 |
| 2017 Kabat | * | * | * | * | * | * | * | * | * | 9 |
| 2017 Park | * | * | * | * | * | * | * | * | * | 9 |
| 2018 Kabat | * | * | * | * | * | * | * | * | * | 9 |
| 2018 Kwon men | * | * | * | * | * | * | * | * | * | 9 |
| 2018 Kwon women | * | * | * | * | * | * | * | * | * | 9 |
| 2020 Hashimoto | * | * | * | * | * | * | * | * | * | 9 |
| 2020 Cho | * | * | * | * | * | * | * | / | * | 8 |
| 2020 Chung | * | * | * | * | * | * | * | * | * | 9 |
| 2020 Kim BCa | * | * | * | * | * | * | * | * | * | 9 |
| 2020 Kim PCa | * | * | * | * | * | * | * | * | * | 9 |

REC= representativeness of the cohort, SNEC= selection of the none posed cohort, AE= ascertainment of exposure, DO= demonstration that outcome of interest was not present at start of study, SC= study controls most important factors such as age, AF= study controls for other important factors, AO= assessment of outcome, FU= follow-up long enough for outcomes to occur ('long enough' is defined as 5 year (median)), AFU=adequacy of follow-up of cohort (≥ 80%). "*" means that the study is satisfied the item, and "/" means not.

# ****Table s3. Incidence rate of cancer in selected studies.****

| Study | Follow-up duration | MHO | | | MUO | | |
| --- | --- | --- | --- | --- | --- | --- | --- |
|  |  | Events | N | IR | Events | N | IR |
| 2010 Arnlov | median: 30 years | 13 | 30 | 14.444 | 21 | 66 | 11.616 |
| 2016 Murphy | median: 3.7 years | 93 | 214 | 117.454 | 385 | 737 | 141.186 |
| 2017 Kabat | 15 years (Overall) | 202 | 3347 | 4.0235 | 345 | 4902 | 4.6920 |
| 2017 Park | mean: 6.4 years | 181 | 6014 | 5.3834 | 721 | 20966 | 6.4089 |
| 2018 Kabat | 15 years (Overall) | 70 | 4038 | 1.1557 | 110 | 4931 | 1.4872 |
| 2018 Kwon men | median: 5.3 years | 91 | 11700 | 1.5 | 394 | 49315 | 1.5 |
| 2018 Kwon women | median: 5.3 years | 61 | 3702 | 3.1 | 245 | 9569 | 4.8 |
| 2020 Hashimoto | median: 5.5 years | 1 | 653 | 0.25 | 25 | 3425 | 1.21 |
| 2020 Cho | 2009-2015 | 567 | 28557 | - | 2009 | 86238 | - |
| 2020 Chung | median: 6.1 years | 133 | 65983 | 0.34 | 172 | 54349 | 0.53 |
| 2019 Kim | median: 5.4 years | 7199 | 2312838 | 0.57422 | 12608 | 2067004 | 1.13926 |
| 2020 Kim | median: 5.4 years | 2193 | 2313991 | 0.17467 | 4045 | 2069401 | 0.36444 |

IR = Incidence Rate (per 1000 person-years); MHO = Metabolically Healthy Obesity; MUO = Metabolically Unhealthy Obesity

Figure s1. Publication bias assessment by Egger’s test and Begg’s test.


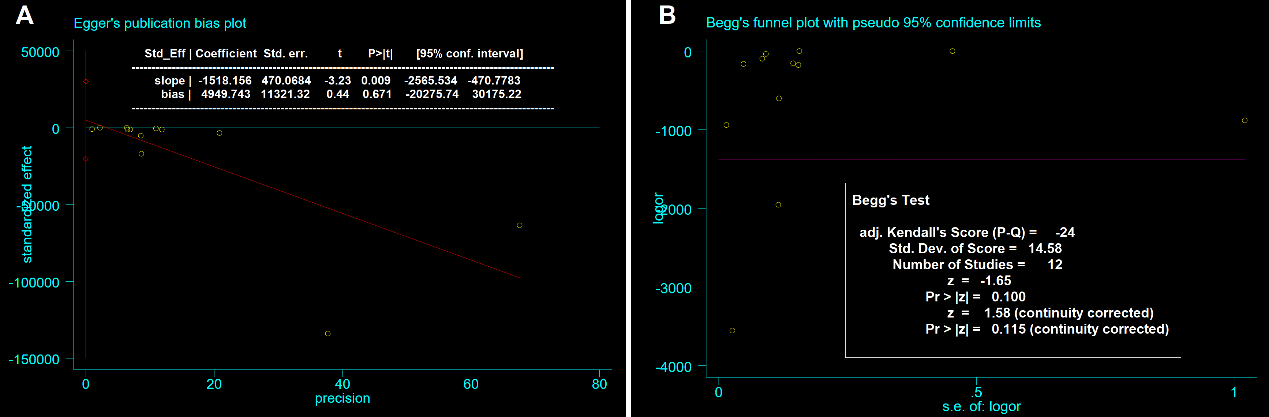


Figure s2. Heterogeneity test to reveal the cause of heterogeneity by excluding certain studies**.** Test A excluded three studies and found the heterogeneity reduced to I^2^ = 28%; Test B excluded two studies and found the heterogeneity reduced to I^2^ = 45%;


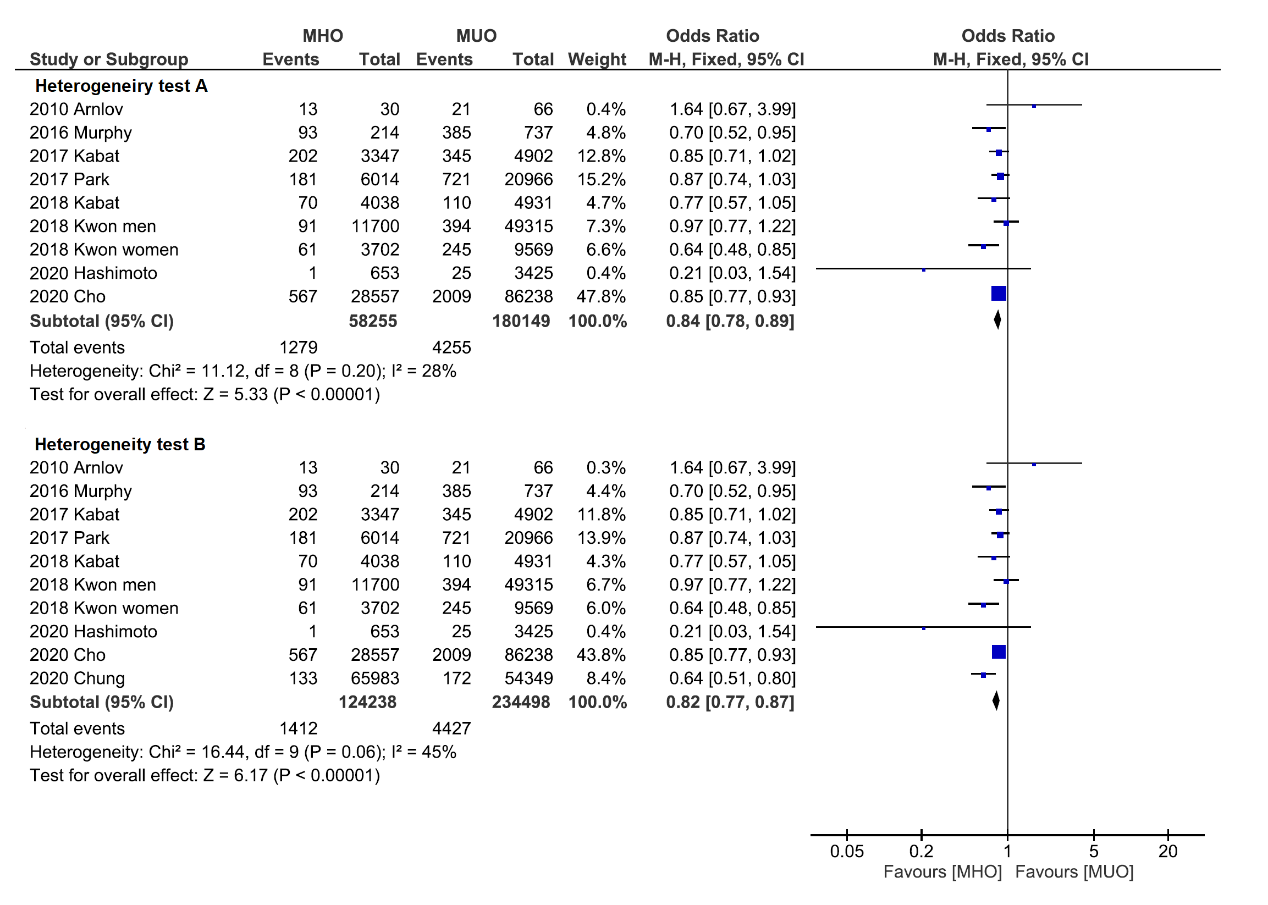
 **Figure s3. Subgroup analysis categorized by the definition of obesity.** Pooled outcome of studies defining BMI >30 as obesity indicates that there is no significant difference of cancer incidence between the MHO and MUO group, but the subgroup defining BMI >25 as obesity showed that MHO has lower incidence of cancer.


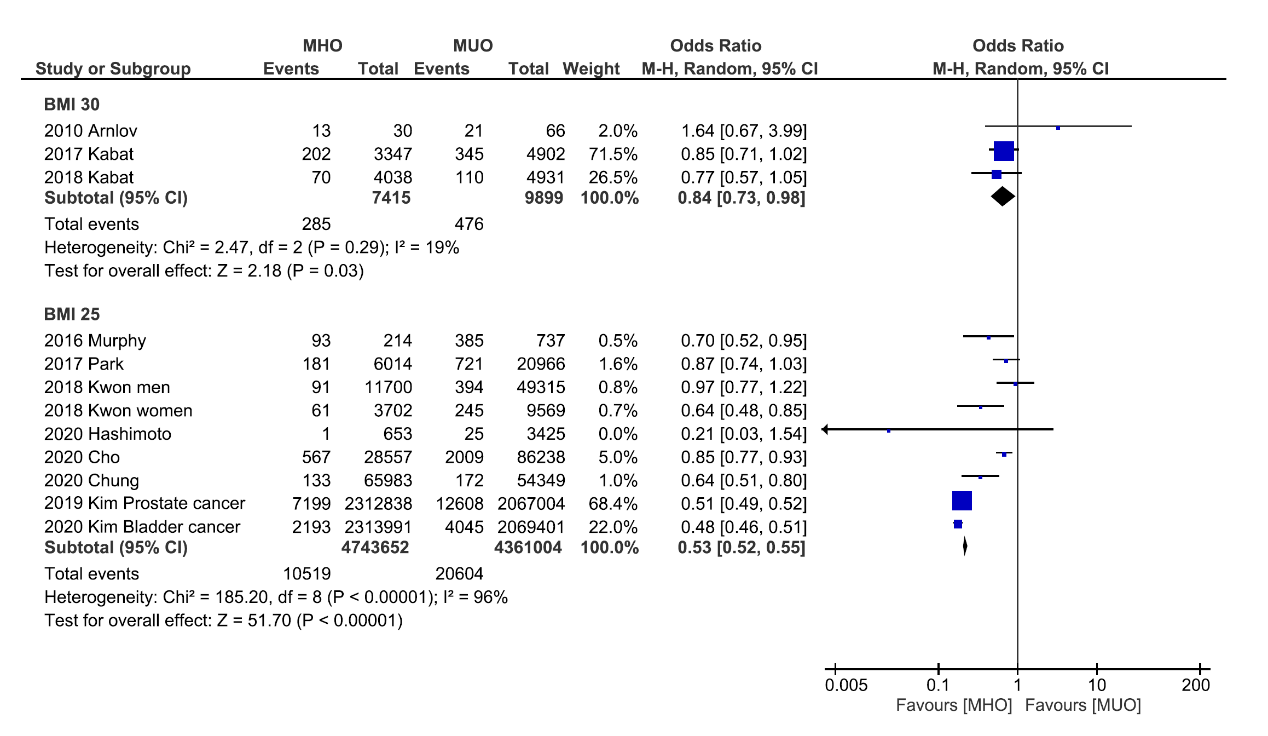


Figure s4. Forest plots comparing the incidence of cancer between different groups (MHO, MUO and MUNW) versus MHNW using the reported odds ratios**.** A) Pooled OR indicates that MHO has higher cancer incidence than MHNW; B) Pooled OR indicates that MUO has higher cancer incidence than MHNW; C) Pooled OR indicates that MUNW has higher cancer incidence than MHNW;


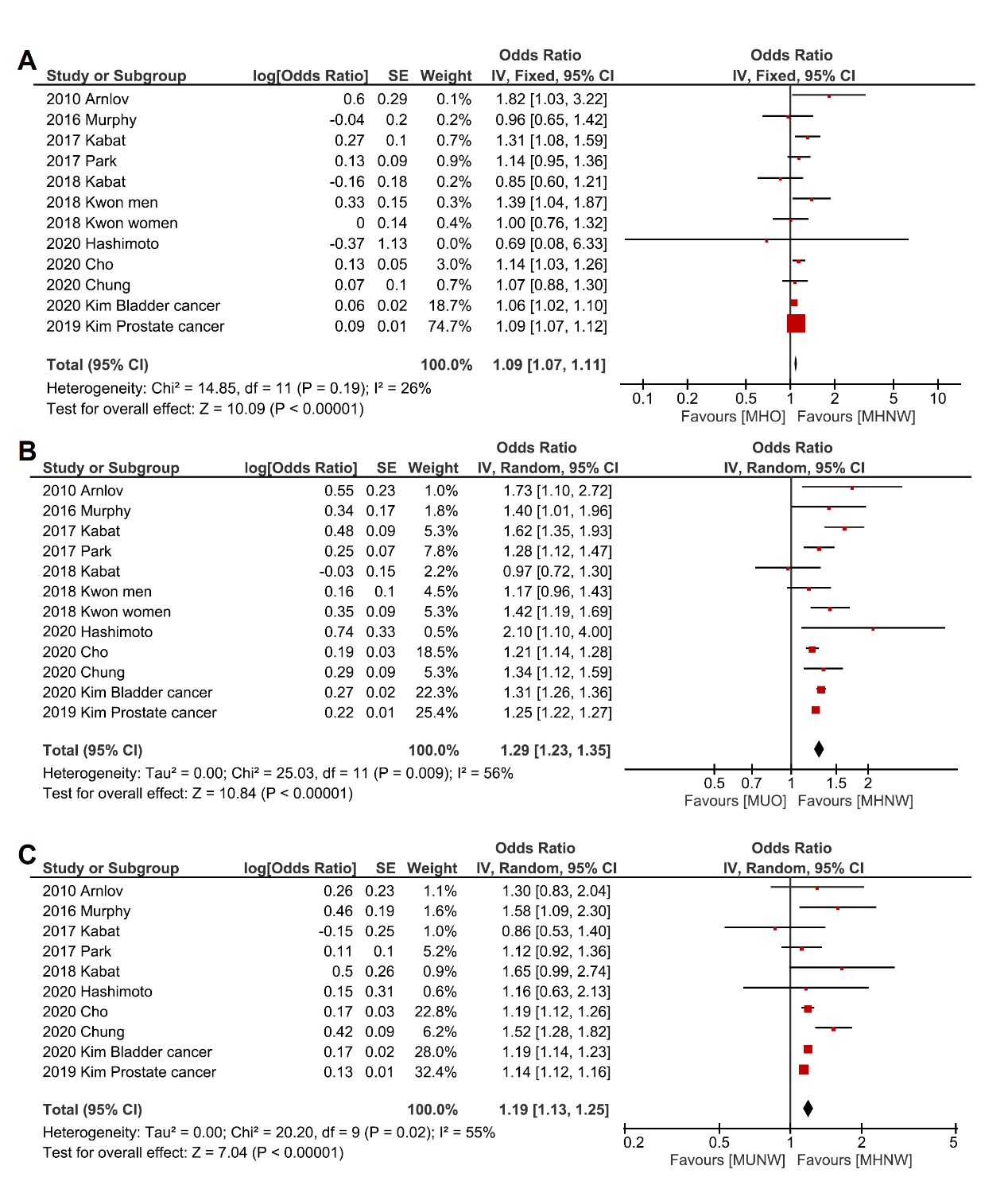


# Figure s5. Funnel plot for studies comparing the incidence of cancer between MHO and MHNW groups.


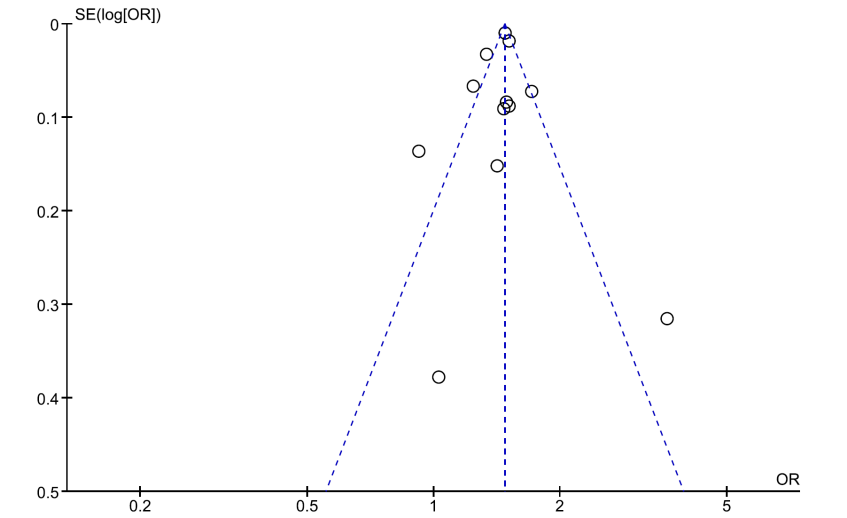


**Figure s6.** Funnel plot for studies comparing the incidence of cancer between MUO and MHNW groups.


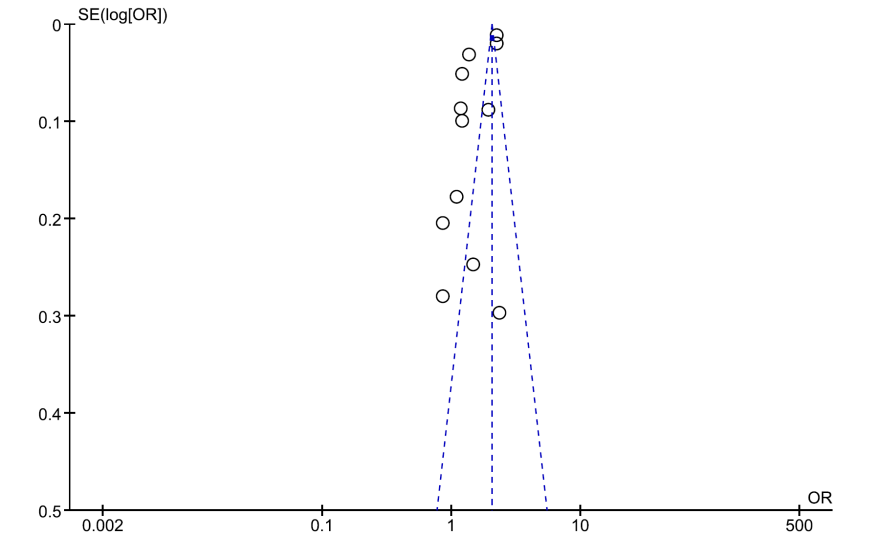


**Figure s7.** Funnel plot for studies comparing the incidence of cancer between MUNW and MHNW groups.


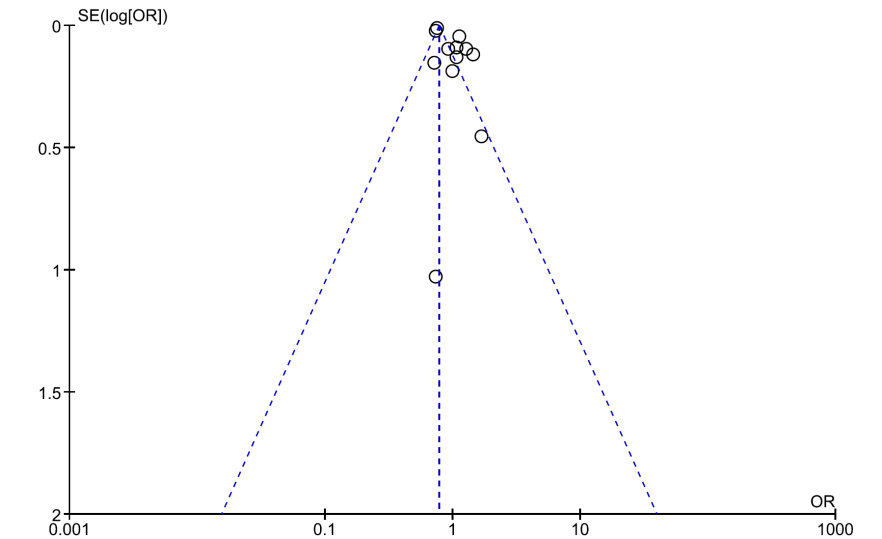

Supplement: Supplementary file 1 [file Data_Sheet_1.docx]
